# Molecular profiling of colorectal cancer in a genetically admixed Hispanic population

**DOI:** 10.1002/cam4.5888

**Published:** 2023-04-11

**Authors:** Ingrid M. Montes‐Rodríguez, Hilmaris Centeno‐Girona, Camila Rivera‐Lynch, Noridza Rivera, Marcia Cruz‐Correa

**Affiliations:** ^1^ Division of Cancer Biology University of Puerto Rico Comprehensive Cancer Center San Juan Puerto Rico USA; ^2^ Department of Medicine University of Puerto Rico School of Medicine San Juan Puerto Rico USA

**Keywords:** colorectal cancer, GENIE, Hispanics, molecular profiling, precision oncology, TCGA

## Abstract

**Backgorund:**

Colorectal cancer (CRC) is among the leading causes of cancer‐related deaths among Hispanics living in the United States (USH). Understanding the most common carcinogenic molecular pathways that affect Hispanics with CRC is crucial to guide research efforts in developing new therapeutic modalities incorporating genomically diverse populations. Tumor profiling techniques help identify actionable alternatives to recommend treatment and improve survival in cancer patients.

**Methods:**

We conducted a secondary data analysis to evaluate the mutational profile of 218 CRC tumors in Hispanics living in Puerto Rico (PRH) who underwent next‐generation sequencing (NGS) testing from 2015 to 2020. We compared the prevalence of CRC tumor somatic mutations in PRHs with the mutational profiles reported for CRC from The Cancer Genome Atlas (TCGA) Pan‐Cancer Clinical Data, the AACR Project Genomics Evidence Neoplasia Information Exchange (GENIE)‐Non‐Hispanic, and GENIE‐Hispanic datasets.

**Results:**

Among the top mutated genes in CRC tumors in PRHs were *APC*, *TP53*, and *KRAS*, which had significantly higher mutational frequencies in PRH compared to the examined datasets, including GENIE‐Hispanics. The most frequent gene amplifications for PRH were *CDX2, CDKN1B*, and *HNRNPA2B1*. Targetable biomarkers for CRC, such as microsatellite instability‐high (MSI), wild‐type *KRAS*, wild‐type *NRAS*, V600E *BRAF*, and *ERBB2* gene amplifications were found in 2.0%, 43.8%, 97.8%, 3.9%, and 2.3%, respectively, of PRH patients.

**Conclusion:**

This is the first study to report the mutational profile of CRC tumors in PRHs and make comparisons to other non‐Hispanic and USH populations.

## 1 INTRODUCTION

Colorectal cancer (CRC) is the third most common cancer diagnosed in men and women in the United States (US) and the third leading cause of cancer‐related deaths.^1^ CRC is a highly heterogeneous disease with different clinicopathological manifestations and clinical outcomes among racial/ethnic populations.^2^ Furthermore, CRC incidence and mortality rates have differed consistently among other racial/ethnic groups, including Hispanic subpopulations. Among Hispanics living in the mainland United States (USH), CRC is the second and fourth most commonly diagnosed cancer for men and women, respectively, and the third‐leading cause of cancer‐related deaths for both sexes.^3^ However, the term Hispanic aggregates various subpopulations, masking the significant variability within subgroups regarding CRC incidence and mortality.^4^ For example, Cubans and Puerto Ricans living in the United States have disproportionately higher CRC incidence and mortality rates than other USH subgroups.^5^, ^6^ For Hispanics living in Puerto Rico (PRH), CRC is the second most diagnosed cancer for both sexes and is the leading cause of cancer‐related deaths.^7^ Overall, PRHs have a higher incidence/mortality rate due to CRC than USHs.^8^

The genetic makeup of Hispanics exhibits a complex population structure, arising from more than 500 years of genetic admixture with varied proportions of African, European, and Native American ancestry.^9^ Moreover, mutational differences in genes that promote tumor progression among different ethnic groups may be important in CRC heterogeneity observed among Hispanic populations.^2^ This suggests that genetic factors may play a significant role in CRC disparities, underscoring the need to characterize the molecular features of admixed populations to develop effective targeted therapies to impact clinical outcomes. This study describes the mutational profile of CRC tumors from PRH, the second‐largest Hispanic group in the United States.^10^ Additionally, we compared the mutational frequency of actionable and driver genes in CRC tumorigenesis for PRH with other non‐Hispanic and USH populations. Together, these datasets highlight the molecular mechanisms underpinning these biological differences.

Most CRC cases arise from somatic mutations, but approximately 5%–10% are due to germline mutations.^11^ Sporadic CRC tumors arise heterogeneously from the accumulation of genetic and epigenetic alterations leading to the transformation of adenomas into adenocarcinomas. A multi‐step sequence for CRC tumorigenesis has been proposed where alterations in genes involved in cellular pathways, such as EGFR (ERBB1/HER1), MAPK, PI3K, TGF‐β, and WNT/β‐catenin signaling pathways, occur at different stages of tumor progression.^12^, ^13^ Descriptive information regarding the genetic mutational frequency of colorectal tumors among Hispanics using next‐generation sequencing (NGS) technologies is limited. We present for the first time a comprehensive somatic molecular characterization of CRC from PRH and compared it to three independent cohorts of patients with CRC from The Cancer Genome Atlas (TCGA) dataset^14^ and AACR Project Genomics Evidence Neoplasia Information Exchange Non‐Hispanics (GENIE‐NH) and Hispanics (GENIE‐H) datasets.^15^ By evaluating colorectal tumors from molecularly characterized ethnically diverse cohorts, we hope to gain a better understanding of the common carcinogenic molecular pathways that affect our Hispanic population. The characterization of the oncogenic drivers in Hispanics will inform clinicians, scientists, and health policy stakeholders about actionable mutations to guide research, treatment, and health policy efforts.

## 2 METHODOLOGY

Using a cross‐sectional design, we evaluated CRC tumors' mutational profiles from 218 PRH that underwent NGS testing from 2015 to 2020. The data were provided by the Precision Oncology Alliance (POA) (https://www.carislifesciences.com/collaboration/) using the CARIS Life Sciences NGS platform, which uses two‐gene panel versions (one containing 592 genes and the other containing 54 genes) to detect mutations, indels, and copy number amplification (CNA). In some samples, genes were ruled indeterminate due to the low coverage of some or all exons. The CNA is calculated using the average sequencing depth of the sample and the sequencing depth of each exon and comparing this result to a pre‐calibrated value. The resulting categories were: (1) *Amplification*: all exons within the gene of interest have an average of ≥3 copies, and the average copy number of the entire gene is ≥6 copies; (2) *Intermediate*: an average of ≥4 but <6 copies of a gene were detected, or if the average copy number of the gene is ≥6 copies, but contains exons with an average of <3 copies; (3) *No amplification* detected: an average of <4 copies of a gene are detected. No clinicopathological data were available for the mutational data provided by the POA. The age and sex data provided cannot be correlated with the mutational data.

To determine the actionable mutations, the OncoKB (https://www.oncokb.org/levels) therapeutic levels of evidence 1, 2, and R1 were considered: (1) FDA‐recognized biomarker predictive response to an FDA‐approved drug; (2) Standard care biomarker recommended by the NCCN or other professional guidelines predictive of response to an FDA‐approved drug; and (R1) Standard care biomarker predictive of resistance to an FDA‐approved drug. Additionally, to classify the gene alterations found for PRH as oncogenic, likely oncogenic, or unknown, we used the OncoKB prior knowledge about specific variants, which contains information about the oncogenic effects and treatment implications or variants.^16^ My Cancer Genome website was used to identify the clinical impact of the observed biomarkers for CRC tumors in PRH and to identify clinical trials that used the observed biomarkers as inclusion criteria for CRC or solid tumors. The information was obtained from FDA labels, the NCCN, and clinical trials, among other scientific platforms.

We estimated the prevalence of somatic mutations in PRH CRC tumors and compared them with the mutational profiles reported for CRC tumors from the TCGA Pan‐Cancer Atlas Clinical Data and CRC tumors from the AACR Project GENIE (public release version 9.0), both available in the cBioPortal for Cancer Genomics. The mutational data reported for the TCGA cohort was based on exome‐sequencing and Affymetrix 6.0 microarrays,^14^ while for the GENIE cohort, the data reported was based on a hybridization‐based or amplicon‐based sequencing approach according to each participating center.^15^ The GENIE database had 724 cases of Hispanics; these cases were taken as an independent subset to compare PRH with the Hispanics in GENIE (GENIE‐H) and the 9427 cases available for GENIE‐Non‐Hispanics (GENIE‐NH).

### 2.1 Statistical analysis

We used descriptive statistics to characterize the datasets. For descriptive purposes, the age variable was dichotomized into <50 and ≥50. However, the mutational data for CRC in PRH were not stratified by age. This variable was reported as age at diagnosis for PRH and TCGA; meanwhile, for GENIE, it represented the age at which sequencing was performed. The equality of the proportions between our study population and the TCGA, GENIE‐NH, and GENIE‐H were evaluated using the two‐sample proportion test (prtesti); a *p*‐value less than 0.05 was considered statistically significant or marginally significant (*p*‐value = 0.05). All statistical analyses were conducted using Stata version 17 for Microsoft Windows (StataCorp LLC). Lollipop graphs using the cBioPortal Mutation Mapper (https://www.cbioportal.org/mutation_mapper) and the GraphPad software were used to visualize t genes and frequency of mutations impacting our Hispanic study population.

## 3 RESULTS

### 3.1 Comparison of the population characteristics of PRH, TCGA, GENIE‐NH, and GENIE‐H datasets

Table 1 compares sex, age at which sequencing was reported, and ethnicity for the PRH, TCGA, and GENIE cohorts. Of the 218 PRH cases, 24.0% had a CRC diagnosis before 50 years of age (early‐onset CRC) since these patients were <50 years old when sequencing was performed. For the PRH population, males accounted for more than half of the sampled population (55.5%), similar to the distribution among the TCGA (52.5%), GENIE‐NH (54.6%), and GENIE‐H (56.1%) datasets. For TCGA CRC tumors, 13.3% were diagnosed before 50 years of age. Like PRH, the GENIE‐NH (27.0%) and GENIE‐H (28.5%) datasets have a higher percentage of sequenced individuals younger than 50.

**TABLE 1:** Socio‐demographic characteristics of PRH, TCGA, GENIE‐NH, and GENIE‐H datasets.

	PRH (*n* = 218)		TCGA (*n* = 594)			GENIE‐NH (*n* = 9427)			GENIE‐H (*n* = 724)		
Characteristics	Cases	%	Cases	%	*p*‐value* (PRH vs. TCGA)	Cases	%	*p*‐value* (PRH vs. GENIE‐NH)	Cases	%	*p*‐value* (PRH vs. GENIE‐H)
Sex											
Men	121	55.5%	312	52.5%	0.447	5143	54.6%	0.792	406	56.1%	0.880
Women	97	44.5%	280	47.1%	0.504	4277	45.4%	0.792	318	43.9%	0.880
Unknown	0	0.0%	2	0.3%	0.389	7	0.1%	0.640	0	0.0%	‐
Age at which sequencing was reported											
Mean	58.5	‐	66.1	‐	‐	58.0	‐	‐	57.9	‐	
15–19	0	0.0%	0	0.0%	‐	17	0.2%	**0.005**	3	0.4%	0.344
20–29	1	0.5%	0	0.0%	0.085	128	0.01	0.260	21	2.9%	0.040
30–39	16	7.3%	16	2.7%	**0.003**	652	6.9%	0.818	53	7.3%	0.992
40–49	35	16.1%	59	9.9%	**0.015**	1747	18.5%	0.366	129	17.8%	0.557
50–59	59	27.1%	96	16.2%	**<0.001**	2500	26.5%	0.843	174	24.0%	0.357
60–69	62	28.4%	166	27.9%	0.899	2513	26.7%	0.575	189	26.1%	0.501
70–79	40	18.3%	160	26.9%	**0.011**	1329	14.1%	0.079	107	14.8%	0.209
80–89	5	2.3%	85	14.3%	**<0.001**	449	4.8%	0.086	33	4.6%	0.137
≥90			10	1.7%	0.054	75	0.8%	0.185	15	2.1%	**0.032**
Unknown	0	0.0%	2	0.3%	0.389	17	0.2%	0.509	0	0.0%	‐
<50	52	23.9%	75	12.6%	**<0.001**	2544	27.0%	0.308	206	28.5%	0.187
≥50	166	76.1%	517	87.0%	**<0.001**	6866	72.8%	0.279	518	71.5%	0.187
Ethnicity											
Hispanic	218	100%	5	0.8%	**<0.001**	‐	0.0%	**<0.001**	724	100%	‐
Non‐Hispanic	0	0.0%	343	57.7%	**<0.001**	7192	76.3%	**<0.001**	0	0.0%	‐
Unknown	0	0.0%	246	41.4%	**<0.001**	2235	23.7%	**<0.001**	0	0.0%	‐

*Note*: Bold indicates statistical significant *p* values.

* *p*‐value was calculated using a test of proportions, <0.05 values are considered statistically significant.

The following sections describe only genes and mutations with a statistically significant difference (*p* < 0.05) when comparing PRH versus TCGA, GENIE‐NH, and GENIE‐H datasets.

### 3.2 Mutational profile of CRC tumors from PRH: Top mutated and actionable genes and comparison with TCGA and GENIE datasets

**FIGURE 1:**
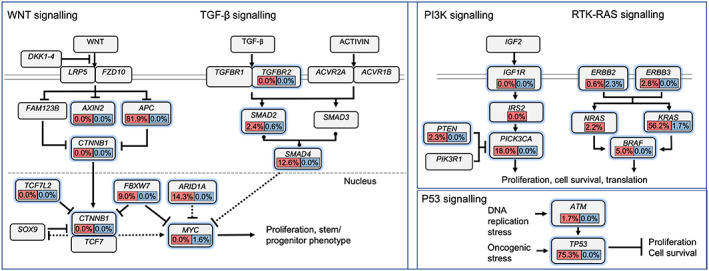
Frequency of genetic alterations in CRC leading to deregulation of the WNT/β‐catenin, MAPK, PI3K, TGF‐β, and p53 signaling pathways. Frequencies of alterations are expressed as a percentage of all cases (values are only reported for profiled genes). The red color denotes the overall mutational frequency, and the blue denotes the amplification frequency for each gene. Figure adapted from “Comprehensive molecular characterization of human colon and rectal cancer” by The Cancer Genome Atlas Nature 2012, 487 (7407), 330–337.

**FIGURE 2:**
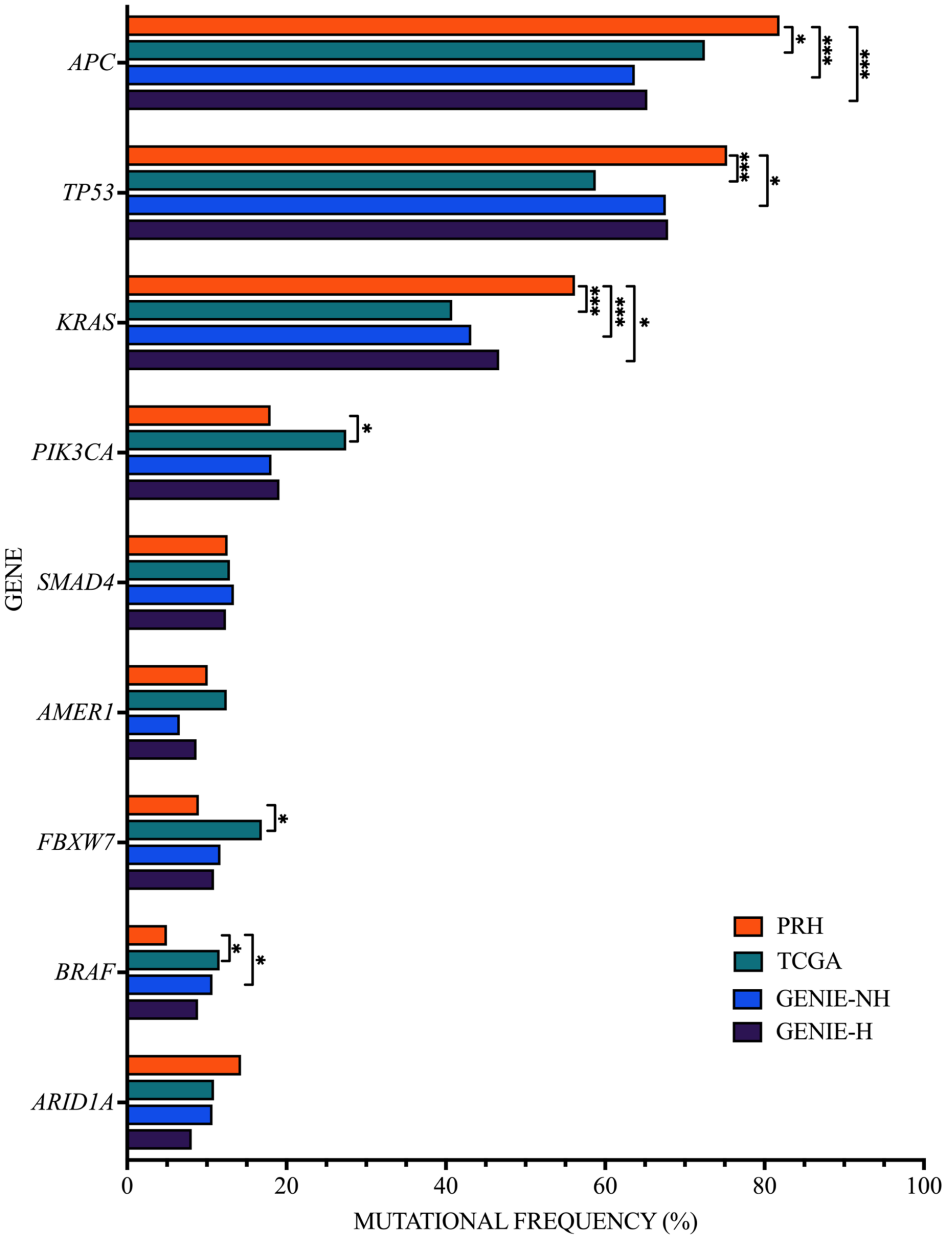
Mutational frequencies of *APC, TP53, KRAS, PIK3CA, SMAD4, AMER1, FBXW7, BRAF*, and *ARID1A* in PRH, TCGA, GENIE‐NH, and GENIE‐H. Significant differences between PRH and other datasets are denoted with an asterisk (*p < 0.05, **p < 0.01, and ***p < 0.001).

The most commonly mutated genes among CRC tumors for PRH were *APC, TP53, KRAS, PIK3CA*, *SMAD4*, *AMER1*, *FBXW7*, *BRAF*, and *ARID1A*. Table 2 includes the top mutated genes in CRC tumors from PRH, along with their most frequent gene‐specific alterations alongside those reported in the TCGA, GENIE‐NH, and GENIE‐H datasets. Figure 1 shows the frequency of the most common genetic alterations found in CRC tumors for PRH leading to deregulation of the WNT/β‐catenin, MAPK, PI3K, TGF‐β, and p53 signaling pathways. Figure 2 shows the mutational frequencies of these genes for PRH compared to other datasets. Only 2.0% of the 150 PRH‐profiled tumors were microsatellite instability‐high (MSI‐H). A comparison between the frequency of MSI tumors from PRH and the TCGA and both GENIE datasets was not performed since this information was not available for these datasets. In this section, we will discuss only those genes that had significant differences when comparing the mutational frequencies of PR with other datasets.

**TABLE 2:** Comparison of the mutational frequencies of most mutated genes and actionable genes found in PRH CRC with those reported in TCGA, GENIE, and GENIE‐H datasets.

		PR			TCGA				GENIE‐NH				GENIE‐H			
Gene	Gene Alteration (OncoKB annotation^a^)	Samples w/ alterations	Profiled tumors	Frequency	Samples w/ alterations	Profiled tumors	Frequency	*p*‐value* (PRH vs. TCGA)	Samples w/ alterations	Profiled tumors	Frequency	*p*‐value* (PRH vs. GENIE‐NH)	Samples w/ alterations	Profiled tumors	Frequency	*p*‐value* (PRH vs. GENIE‐H)
*APC*	*Overall*	149	182	81.9%	387	534	72.5%	**0.012**	5837	9159	63.7%	**<0.001**	454	695	65.3%	**<0.001**
	E1309* (LO)	16	182	8.8%	3	534	0.6%	**<0.001**	72	9159	0.8%	**<0.001**	5	695	0.7%	**<0.001**
	E1309fs (LO)	11	182	6.0%	0	534	0.0%	**<0.001**	274	9159	3.0%	**0.018**	17	695	2.4%	**0.015**
	T1556fs (LO)	8	182	4.4%	11	534	2.1%	0.097	201	9159	2.2%	**0.046**	14	695	2.0%	0.067
	T1493fs (LO)	5	182	2.7%	4	534	0.7%	**0.033**	24	9159	0.3%	**<0.001**	0	695	0.0%	**<0.001**
	R1450* (LO)	4	182	2.2%	34	534	6.4%	**0.029**	408	9159	4.5%	0.129	41	695	5.9%	**0.044**
*TP53*	*Overall*	134	178	75.3%	314	534	58.8%	**<0.001**	6279	9289	67.6%	**0.030**	489	720	67.9%	**0.055**
	R175H (O)	12	178	6.7%	32	534	6.0%	0.737	612	9289	6.6%	0.936	45	720	6.3%	0.825
	R282W (LO)	8	178	4.5%	20	534	3.7%	0.633	356	9289	3.8%	0.650	34	720	4.7%	0.900
	G245S (O)	7	178	3.9%	6	534	1.1%	**0.015**	180	9289	1.9%	0.059	16	720	2.2%	0.204
	R342* (LO)	4	178	2.2%	3	534	0.6%	0.063	135	9289	1.5%	0.379	4	720	0.6%	**0.036**
	P278S (LO)	2	178	1.1%	0	534	0.0%	**0.015**	16	9289	0.2%	**0.004**	0	720	0.0%	**0.005**
*KRAS* ^b^	*Overall*	104	185	56.2%	218	534	40.8%	**<0.001**	4076	9427	43.2%	**<0.001**	338	724	46.7%	**0.021**
	G12D (O)	29	185	15.7%	59	534	11.0%	0.098	1200	9427	12.7%	0.234	100	724	13.8%	0.515
	G13D (O)	24	185	13.0%	37	534	6.9%	**0.011**	684	9427	7.3%	**0.003**	63	724	8.7%	0.078
	G12C (O)	13	185	7.0%	15	534	2.8%	**0.011**	282	9427	3.0%	**0.002**	20	724	2.8%	**0.006**
	G12V (O)	12	185	6.5%	49	534	9.2%	0.258	824	9427	8.7%	0.282	73	724	10.1%	0.134
	Q61H (O)	5	185	2.7%	4	534	0.7%	**0.040**	99	9427	1.1%	**0.032**	6	724	0.8%	**0.038**
*PIK3CA*	*Overall*	32	178	18.0%	147	534	27.5%	**0.011**	1706	9424	18.1%	0.967	138	724	19.1%	0.737
	E545K (O)	13	178	7.3%	35	534	6.6%	0.730	420	9424	4.5%	0.071	37	724	5.1%	0.253
*SMAD4*	*Overall*	22	175	12.6%	69	534	12.9%	0.918	1234	9197	13.4%	0.744	89	717	12.4%	0.943
	R361S (LO)	2	175	1.1%	0	534	0.0%	**0.015**	13	9197	0.1%	**0.001**	0	717	0.0%	**0.005**
*AMER1*	*Overall*	17	169	10.1%	67	534	12.5%	0.401	292	4398	6.6%	0.082	27	309	8.7%	0.613
	R358* (LO)	3	169	1.8%	4	534	0.7%	0.202	16	4398	0.4%	**0.005**	3	309	1.0%	0.438
	R641* (LO)	2	169	1.2%	0	534	0.0%	**0.011**	9	4398	0.2%	**0.010**	3	309	1.0%	0.815
*FBXW7*	*Overall*	16	177	9.0%	90	534	16.9%	**0.011**	1077	9197	11.7%	0.273	78	716	10.9%	0.461
	R465C (O)	3	177	1.7%	9	534	1.7%	0.999	122	9197	1.3%	0.680	9	716	1.3%	0.647
	R505C (O)	2	177	1.1%	5	534	0.9%	0.812	109	9197	1.2%	0.942	9	716	1.3%	0.865
	R465H (LO)	2	177	1.1%	12	534	2.2%	0.356	108	9197	1.2%	0.961	10	716	1.4%	0.758
*BRAF* ^b^	*Overall*	9	180	5.0%	62	534	11.6%	**0.011**	1013	9424	10.7%	**0.013**	64	723	8.9%	0.087
	V600E (O)	7	180	3.9%	48	534	9.0%	**0.027**	723	9424	7.7%	0.058	45	723	6.2%	0.230
	D594G (O)	1	180	0.6%	0	534	0.0%	0.084	65	9424	0.7%	0.834	4	723	0.6%	0.987
	F595L (O)	1	180	0.6%	0	534	0.0%	0.084	3	9424	0.0%	**<0.001**	1	723	0.1%	0.286
*ARID1A*	*Overall*	6	42	14.3%	58	534	10.9%	0.500	706	6618	10.7%	0.449	31	381	8.1%	0.176
	Q1519fs (LO)	1	42	2.4%	0	534	0.0%	**<0.001**	16	6618	0.2%	**0.006**	1	381	0.3%	0.056
	K1072fs (LO)	1	42	2.4%	2	534	0.4%	0.090	6	6618	0.1%	**<0.001**	1	381	0.3%	0.056
*NRAS* ^b^	*Overall*	4	181	2.2%	33	534	6.2%	**0.037**	428	9423	4.5%	0.134	36	723	5.0%	0.105
	G13D (O)	2	181	1.1%	1	534	0.2%	0.102	13	9423	0.1%	**0.001**	2	723	0.3%	0.138
	Q61K (O)	1	181	0.6%	9	534	1.7%	0.260	98	9423	1.0%	0.518	6	723	0.8%	0.701
	Q61L (O)	1	181	0.6%	1	534	0.2%	0.429	22	9423	0.2%	0.380	5	723	0.7%	0.835
*ERBB2* ^**b**^	*Overall*	1	178	0.6%	20	534	3.7%	**0.029**	329	9418	3.5%	**0.034**	34	723	4.7%	**0.010**
	V777L (O)	1	178	0.6%	2	534	0.4%	0.733	24	9418	0.3%	0.415	0	723	0.0%	**0.044**
*NTRK1* ^b^	*Overall*	0	171	0.0%	9	534	1.7%	0.087	174	6647	2.6%	**0.032**	10	406	2.5%	**0.039**
*NTRK3* ^b^	*Overall*	0	169	0.0%	22	534	4.1%	**0.007**	217	6564	3.3%	**0.0162**	12	380	3.2%	**0.020**

*Note*: Bold indicates statistical significant *p* values.

^a^ OncoKB annotation: Likely Oncogenic (LO), Unknown (Unk).

^b^ Actionable genes for Colorectal Cancer.

* *p*‐value was calculated using a test of proportions, <0.05 values are considered statistically significant.

#### 3.2.1 APC

**FIGURE 3:**
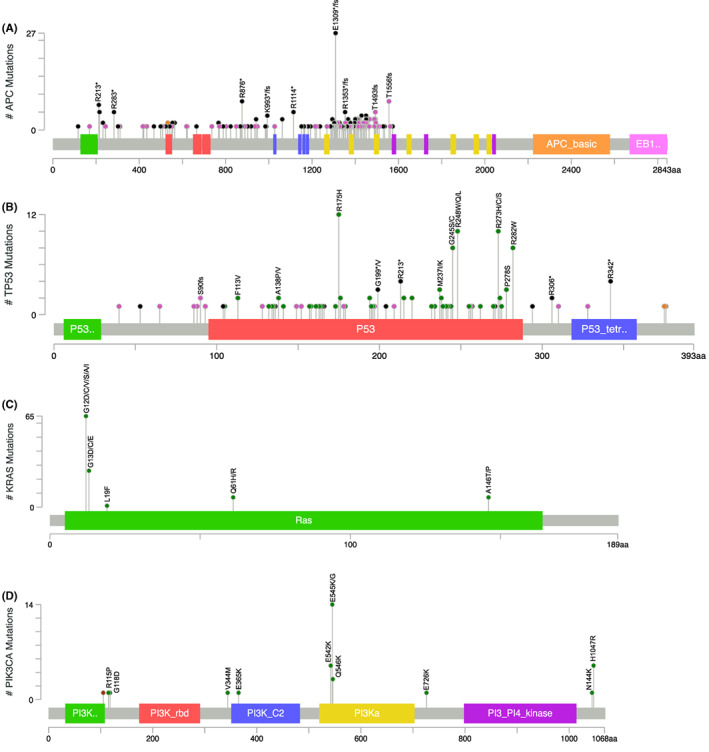
Lollipop plots show the distribution and classes of mutations in the top four most mutated genes for CRC tumors from PRH. Any position with a mutation is represented as a circle, and the line length depends on the number of mutations detected at that codon. Frameshifts, nonsense, and missense mutations are represented in pink, black, and green, respectively; splice variants are represented in orange. The gray bar represents the entire protein with the different amino acid positions (aa). The colored boxes are specific functional domains. On top of the lollipops, the most frequent variants are annotated as the amino‐acid change at that specific site. Mutations were identified in the (A) *APC* gene using NGS, (B) *TP53*, (C) *KRAS*, and (D) *PIK3CA*.

The *APC* mutational frequency for PRH was 9.4% higher than TCGA, 18.1% higher than GENIE‐NH, and 16.5% higher than GENIE‐H. In terms of specific gene alterations, 134 unique gene alterations were reported for the *APC* gene: 132 (98.5%) were truncating mutations (including 68 frameshift and 64 nonsense mutations) and two splice variants (1.5%). Figure 3 shows the mutational distribution of *APC* for PRH. The most frequent *APC* gene alteration for PRH was E1309*—8.2%, 8.0%, and 8.1% higher than that reported for TCGA, GENIE‐NH, and GENIE‐H, respectively. The second most frequent gene alteration for PRH was E1309fs, which was not reported for TCGA, and lower frequencies were reported for GENIE‐NH and GENIE‐H. For PRH, the frequency of T1556fs was 2.2% higher than for GENIE‐NH, while T1493fs was 2.0%, 2.5%, and 2.8% higher than those for TCGA, GENIE‐NH, and GENIE‐H, respectively. PRH had a lower frequency of R1450*, 4.2% and 3.7% lower than TCGA and GENIE‐H, respectively.

#### 3.2.2 TP53

PRH had the highest *TP53* mutational frequency among the compared datasets: 16.5% higher than TCGA, 7.7% higher than GENIE‐NH, and 7.4% higher than GENIE‐H. A total of 88 unique gene alterations were reported for the *TP53*, of which 51 (58.0%) were missense mutations, 28 (31.8%) truncating mutations (including 15 frameshift and 13 nonsense mutations), 7 (8.0%) splice variant mutations, and 2 (2.3%) insertion/deletion mutations (see Figure 3). In *TP53*, R175H and R282W were the most frequent variants for PRH, which match the frequencies in the TCGA, GENIE‐NH, and GENIE‐H datasets. G245S was the third most frequent alteration among PRH, with a frequency 2.8% higher than that reported for TCGA. PRH had a 1.6% higher frequency of R342* than that reported for GENIE‐H and a higher frequency of P278S compared to other datasets.

#### 3.2.3 *KRAS* (actionable gene for CRC)

The mutational frequency of *KRAS* for PRH was 15.4% higher than TCGA, 13.0% higher than GENIE‐NH, and 9.5% higher than GENIE‐H. For PRH, 15 different missense mutations were reported for the *KRAS* gene. Figure 3 shows the frequencies of *KRAS* alterations. In detail, *KRAS* G12D, G13D, G12C, and G12V were the most frequent gene alterations for PRH. Compared to other datasets, PRH had a 6.1% and 5.7% higher frequency of G13D than that reported for TCGA and GENIE‐NH, respectively, and a 4.2%, 4.0%, and 4.2% higher frequency of G12C than that reported for TCGA, GENIE‐NH, and GENIE‐H, respectively. PRH also had a 2.0%, 1.6%, and 1.9% higher frequency of *KRAS* Q61H when compared to TCGA, GENIE‐NH, and GENIE‐H, respectively.

#### 3.2.4 PIK3CA

The mutational frequency for *PIK3CA* was 9.6% lower for PRH when compared to TCGA. For PRH, a total of 13 unique gene alterations were reported for the *PIK3CA* gene, of which 11 (84.6%) were missense mutations and 2 (15.4%) were insertion/deletion mutations (see Figure 3). For PRH, E545K was the most frequent alteration for *PIK3CA*, having a similar frequency across all the compared datasets.

#### 3.2.5 FBXW7

The mutational frequency for *FBXW7* was 7.8% lower for PRH when compared to that reported for TCGA, and it was similar to the GENIE and GENIE‐H datasets. A total of 12 different gene alterations in *FBXW7* were reported for PRH, of which 6 (50%) were truncating mutations (including one frameshift and five nonsense mutations), 3 (25.0%) missense mutations, and 3 (25.0%) intronic variants. The most frequent alterations reported for PRH were R456C, R505C, and R4965H, which had similar mutational frequencies compared to other datasets.

#### 3.2.6 *BRAF* (actionable gene for CRC)

For PRH, *BRAF* mutational frequency was 6.6% and 5.7% lower than TCGA and GENIE‐NH, respectively, which is comparable to GENIE‐H. This gene has three different gene alterations for PRH: V600E, D594G, and F595L. The frequencies of these alterations among the other datasets were similar, except for F595L, which was significantly lower for PRH when compared to that reported for GENIE‐NH.

#### 3.2.7 *NRAS* (actionable gene for CRC)

PRH had a 4.0% lower mutational frequency for *NRAS* when compared to that reported for TCGA. For PRH, only three different missense mutations were reported: G13D, Q61K, and Q61L. Additionally, G13D frequency was 1% higher than that reported for GENIE‐NH.

#### 3.2.8 ERBB2

For PRH, *ERBB2* mutational frequency was 3.2%, 2.9%, and 4.1% lower than those reported for TCGA, GENIE‐NH, and GENIE‐H, respectively. Only one case for PRH reported the oncogenic missense mutation *V777L*, which was not reported for this gene for GENIE‐H.

#### 3.2.9 *NTRK1* and *NTRK3* (actionable genes for CRC)

No mutations were found for these genes among CRC tumors from PRH. On the contrary, mutations for these genes were reported for TCGA, GENIE‐NH, and GENIE‐H.

### 3.3 Copy number variants: Gene amplification

The most common gene amplifications found for PRH CRC tumors using NGS are presented in Table 3. For PRH, the most frequent gene amplifications were: *CDX2*, which was 13.7% higher than TCGA; *CDKN1B*, which was 6.3% and 6.2% higher than GENIE‐NH and GENIE‐H, respectively; and *HNRNPA2B1*, which was 5.1% higher than TCGA.

**TABLE 3:** Gene amplifications observed for CRC tumors from PRH and comparison with TCGA, GENIE, and GENIE‐H.

	PR			TCGA				GENIE‐NH				GENIE‐H			
Gene	Samples w/ CNA	Profiled tumors	Frequency (%)	Samples w/ CNA	Profiled tumors	Frequency (%)	*p*‐value* (PRH vs. TCGA)	Samples w/ CNA	Profiled tumors	Frequency (%)	*p*‐value* (PRH vs. GENIE‐NH)	Samples w/ CNA	Profiled tumors	Frequency (%)	*p*‐value* (PRH vs. GENIE‐H)
*CDX2*	37	184	20.1%	38	592	6.4%	**<0.001**	Not profiled				Not profiled			
*CDKN1B*	12	183	6.6%	0	592	0.0%	**<0.001**	16	6030	0.3%	**<0.001**	1	319	0.3%	**<0.001**
*HNRNPA2B1*	10	184	5.4%	2	592	0.3%	**<0.001**	Not profiled				Not profiled			
*SS18L1*	6	182	3.3%	40	592	6.8%	0.084	Not profiled				Not profiled			
*ZNF703*	6	183	3.3%	9	592	1.5%	0.131	4	579	0.7%	**0.007**	0	12	0.0%	**0.017**
*FLT3*	5	181	2.8%	37	592	6.3%	0.070	257	6064	4.2%	0.328	12	320	3.8%	0.539
*MNX1*	5	179	2.8%	1	592	0.2%	**0.001**	Not profiled				Not profiled			
*ARFRP1*	4	184	2.2%	43	592	7.3%	**0.011**	31	576	5.4%	0.071	0	11	0.0%	0.619
*CCND2*	4	184	2.2%	16	592	2.7%	0.692	111	6027	1.8%	0.744	9	318	2.8%	0.667
*FGF3*	4	166	2.4%	1	592	0.2%	**0.002**	18	3958	0.5%	**<0.001**	1	248	0.4%	0.066
*FLT1*	4	179	2.2%	32	592	5.4%	0.077	209	5973	3.5%	0.360	11	317	3.5%	0.429
*ERBB2* ^a^	4	177	2.3%	20	592	3.4%	0.452	133	6064	2.2%	0.950	6	320	1.9%	0.785
*RARA*	4	182	2.2%	14	592	2.4%	0.900	45	5973	0.8%	**0.030**	4	317	1.3%	0.445

*Note*: Bold indicates statistical significant *p* values.

^a^ Actionable genes for Colorectal Cancer.

* *p*‐value was calculated using a test of proportions, <0.05 values are considered statistically significant.

Other gene amplifications significantly different between PRH and other datasets were: *ZNF703*, *MNX1*, *ARFRP1*, *FGF3*, and *RARA*. The amplification of the *ERBB2* gene (an actionable mutation for CRC) was similar among all the compared datasets.

## 4 DISCUSSION

Molecular characterization of CRC tumors has resulted in the identification of genetic alterations in cancer driver genes, such as *KRAS, NRAS, BRAF*, and *ERBB2*, which are now used to guide first‐line therapies.^17^ The introduction of targeted therapies, such as monoclonal antibodies (mAb) and small molecules to inhibit tyrosine kinases, has improved overall survival in metastatic CRC (mCRC).^17^ Despite the development of biomarker‐based stratified treatment, there are still marked survival disparities among different racial and/or ethnic groups, including Hispanics,^8^ underscoring the necessity to identify new predictive and prognostic biomarkers for the development of effective treatments. In this study, we analyzed NGS data from PRH CRC tumors and provided a detailed description of their molecular profile. We also compared the mutational profiles reported for CRC tumors from other national and international datasets. Here we only discuss actionable genes and driver genes with overall mutational frequencies significantly different in PRH compared to other datasets. Our goal was to identify possible alterations that may be clinically significant in Hispanics.

Microsatellite instability‐high (MSI‐H) tumors were less frequent in PRH compared to previously reported for African Americans (12%), Hispanics (12%), and Caucasians (14%) in the United States.^18^ In this study, the low frequency of MSI‐H CRC tumors from PRH correlates with what was previously reported by our group, and only 4.3% of tumors from PRH had negative MMR‐protein expression.^19^ According to the literature, MSI varies depending on CRC stage. Approximately 12–20% of MSI has been observed in the early stages, whereas <5% incidence of MSI has been observed in advanced stages.^20^ Tumor stage information was unavailable for CRC tumors from PRH that were used in this study, and no assumptions could be made about tumor stage and MSI status. Currently, MSI‐H status is a biomarker predictive of response to anti–PD‐1/PD‐L1 therapy (see Table 4).^21^

**TABLE 4:** Biomarkers and treatment according to the highest level of evidence according to OncoKB.

Gene/Biomarker	Gene alteration	Level of evidence^a^ or clinical trial	Therapies	Mechanism of action
MSI		Level 1	Pembrolizumab	***Immune checkpoint inhibitors***—inhibit tumor‐induced immunosuppression by blocking the interaction between PD‐1/PD‐L1, or CTLA‐4/B7 pathways to restore T‐cell antitumor immune response. ‐ **Pembrolizumab** (Keytruda): mAB directed against programmed cell death protein 1 (PD‐1). ‐ **Atezolizumab** (Tecentriq): IgG1 mAb that binds PD‐L1 on the T‐cell. ‐ **Nivolumab** (Opdivo): IgG4 mAbthat binds PD‐1 receptor ‐ **Ipilimumab** (Yervoy): IgG1 mAbthat binds CTLA‐4 on the T‐cell surface. ***Vaccines***—activation of adaptive host immune response ‐ **KRAS‐targeted long peptide vaccine**: peptide vaccine containing a mixture of long peptides derived from tumor‐specific mutant forms of the KRAS antige. Stimulates immune response against cells harboring mutant KRAS proteins. ‐ **GRT‐C903** and **GRT‐R904**: are neoantigen cancer vaccines that stimulates host immunity to mount specific cytotoxic T‐lymphocyte response against tumor cells. ***Tyrosine kinase inhibitors**—*inhibition of EGFR and ERBB2/HER2 signaling pathways lead to tumor cell death, inhibition of cell growth, angiogenesis, and metastasis. ‐ **Cetuximab** (Erbitux) and **Panitumumad** (Vectibix): mAbs that specifically bind EGFR. ‐ **Pertuzumab**: mAb that targets subdomain II of ERBB2/HER2. ‐ **Trastuzumab**: mAb that targets ligand‐binding domain IV of ERBB2/HER2. ‐ **Trastuzumab deruxtecan**: mAb against HER2 conjugated with a topoisomerase I inhibitor (deruxtecan). Internalization of deruxtecan leads to DNA damage and apoptotic cell death. ‐ **Ado‐Trastuzumab Emtansine** (Kadcyla^TM^): mAb against ERBB2/HER2 conjugated with DM1(a microtubule inhibitor). Internalization of DM results in cell arrest and death. ***Small molecules*** ‐ **Encorafenib** (Braftovi): kinase inhibitor that specifically targets BRAF V600E. ‐ **Poziotinib**: kinase inhibitor that targets most of EGFR and ERBB2 exon 20 mutations. ‐ **BDTX‐189**: selectively binds and inhibits irreversibly *EGFR* and *ERBB2* mutants. ‐ **Lapatinib**: protein kinase inhibitor that acts on ERBB2 and EGFR. ‐ **Regorafenib**: multi‐kinase inhibitor. ‐ **Sotorasib** (Lumakras/AMG 510) and MRTX849: inhibit *KRAS* G12C by forming a covalent bond with the cysteine residue at position 12.
			Ipilimumab + Nivolumab	
			Nivolumab	
*BRAF*	V600E	Level 1	Encorafenib + Cetuximab	
		Level 2	Encorafenib + Panitumumab	
*ERBB2*	V777L	Clinical trials	Phase II—Fam‐Trastuzumab Deruxtecan (NCT04639219)	
			Phase II—Atezolizumab + Pertuzumab + Trastuzumab (NCT04551521)	
			Phase II—BDTX‐189 (NCT04209465)	
			Phase II—Poziotinib (NCT04172597)	
	Amp	Level 2	Trastuzumab + Pertuzumab	
			Lapatinib + Trastuzumab	
		Clinical trials	Phase II‐ Ado‐Trastuzumab Emtansine (NCT02675829)	
*KRAS/ NRAS*	Wild Type	Level 1	Cetuximab/ Panitumumab/ Regorafenib	
	G12D/C/V/S/A/I G13D Q61H/R/K/L	Clinical trials	Phase I/II—Sotorasib (NCT03600883)	
			Phase I—Ipilimumab + Nivolumab + KRAS‐Targeted Long Peptide Vaccine (NCT04117087)	
			Phase I—Sotorasib + EGFR inhibitor ± Chemotherapeutic/ MEK inhibitor + EGFR inhibitor (NCT04185883)	
			Phase I/II—GRT‐C903 + GRT‐R904 + Ipilimumab + Nivolumab (NCT03953235)	
			Phase II—MRTX849 + Cetuximab (NCT04793958)	
		Level R1	Predictive resistance to Cetuximab & Panitumumab	

*Note*: Clinical Trials—clinical trials for CRC or solid malignant tumors in which the specified biomarker serves as an inclusion eligibility criterion. Abbreviation: mCRC‐metastatic CRC.

^a^ OncoKB Levels of Evidence V2: Level 1—FDA‐recognized biomarker predictive of response to an FDA‐approved drug in CRC; Level 2—Standard care biomarker recommended by the NCCN or other expert panels predictive of response to an FDA‐approved drug in CRC; Level R1—Standard of care biomarker predictive of resistance to an approved drug in this indication.

Significant differences in the mutational frequencies of CRC driver genes, such as *APC, TP53*, *PIK3CA, FBXW7*, *CDX2*, *CDKN1B*, and *HNRNPA2B1*, were reported for PRH when compared to the datasets examined. Most of these driver genes are members of the WNT/β‐catenin, MAPK, PI3K, TGF‐ β, and p53 pathways (see Figure 1). PRH had the highest mutational frequency for *APC*, a tumor suppressor gene involved in the regulation of the WNT/β‐catenin signaling pathway, and mutations in this gene occur as an early event in CRC tumorigenesis.^22^ Previous studies showed lower *APC* mutational frequency for Caucasians (15.5%), Asians (25.2%), and African Americans (30.9%).^23^ However, the mutational frequency of *APC* for PRH was similar to the Brazilian population (71.4%).^24^ The marked differences in *APC* mutational frequency among populations may reflect on the molecular pathways that lead to tumor progression, suggesting that for PRH, the most common one is the CIN pathway. Even though *APC* is not an actionable gene for CRC treatment, in vitro studies have shown that small‐molecule inhibitors targeting APC truncated proteins either restore the WNT/β‐catenin signaling pathway or cause cytotoxicity.^25^, ^26^ Similarly, PRH had the highest *TP53* mutational frequency among the compared datasets. *TP53* tumor suppressor gene mutations occur as late events in CRC tumorigenesis and are mostly associated with metastasis and poor overall survival.^27^ Considering the high mutational burden of *APC* and *TP53* for PRH, this population will benefit greatly from targeted therapies against mutations in these genes. Moreover, ongoing clinical trials (see Table S1) evaluating new treatment approaches based on these biomarkers could result in new biomarker‐based therapies that will benefit Hispanic populations with a high mutational burden for these genes.

In this study, the mutational frequencies of the *PIK3CA* and *FBXW7* genes were lower for PRH. However, PRH has a higher mutational frequency than those previously reported for other minority groups, including African Americans (13%) and Asian/Pacific Islanders (10.0%).^28^ *PIK3CA* mutations play a key role in CRC pathogenesis, with an incidence of 20% in CRCs.^17^, ^22^ For PRH, most of the mutations were found in *PIK3CA* mutational hot spots, located in exons 9 and 20, which have a distinct molecular impact on CRC: exon 9 mutations are associated with *KRAS* mutations, and exon 20 mutations are associated with *KRAS, BRAF* mutations, and MSI.^29^ *PIK3CA* mutations may predict resistance to first‐line chemotherapy, anti‐EGFR therapy, and response to radioembolization therapy in mCRCs.^30^ Even though contradictory findings assessing the prognostic value of *PIK3CA* mutations in CRC, targeting the PI3K/AKT1 pathway has emerged as a therapeutic alternative for CRC.^31^ Mutations in *FBXW7*, a tumor suppressor gene that downregulates transcriptional activators involved in cell growth, are found in approximately 6%–10% of CRC.^32^ Previous studies have shown that mCRC patients with *FBXW7* mutations have a lower overall survival rate than those with the wild‐type gene.^32^ The mutational frequency of *FBXW7* reported for PRH in this study is consistent with those reported for Asians (6.5%), African‐Americans (8.0%), Whites (7.6%), and Hispanics (7.4%).^32^ Even though multiple studies describe the predictive/prognostic value of these biomarkers in CRC‐targeted therapies and overall survival, there is no significant evidence to recommend selected treatments for mutations in *PIK3CA* and *FBXW7*. However, clinical trials use these biomarkers as inclusion criteria; see Table S1.

PRH had higher mutational frequencies for gene amplification in *CDX2*, *CDKN1B*, and *HNRNPA2B1*. A reduced expression of *CDX2*, a transcriptional regulator of intestinal cell lineage observed in approximately 20% of CRCs, is associated with poor outcomes and has been identified as a prognostic biomarker in stage II/III CRC.^33^ In this study, we reported that PRH had a three times higher frequency for *CDX2* gene amplification when compared to TCGA. *CDX2* amplification is associated with the activation of the Wnt/β‐catenin signaling pathway, playing a role as a lineage‐survival oncogene in CRC.^34^ The role of *CDX2* expression in CRC may rely on the underlined molecular pathways leading to carcinogenesis,^34^ which implies that CRC tumors from PRH are molecularly distinct from other populations. PRH had six and five times more *CDKN1B* and *HNRNPA2B1* gene amplification, respectively, compared to the other datasets. For CRC, it has been reported that the absence or reduction of *CDKN1B* expression is associated with a poor prognosis.^35^ In contrast, *CDKN1B* gene amplification is associated with poor prognosis for gastric carcinomas.^36^ High *CDKN1B* expression predicts sensitivity to hormone therapies and chemotherapy in luminal breast cancer patients, while its downregulation predicts resistance to radiotherapy and anti‐ERBB2 therapies.^37^ *HNRNPA2B1* amplification correlates with a higher gene expression observed in several cancers and is associated with tumor progression and poor prognosis.^38^ Similarly, a recent study reported that high expression of *HNRNPA2B1* in late‐stage CRC (stages III and IV) was associated with tumor progression, metastasis, and poor prognosis.^39^ Further studies are needed to investigate the role of these gene amplifications in CRC carcinogenesis and how they correlate with the high mortality rates observed in PRH.

We also compared the mutational frequencies of actionable genes for CRC and reported significant differences for PRH in *KRAS/NRAS*, *BRAF*, and *ERBB2* amplification. The high mutational frequency of *KRAS* reported for PRH differs from a previous study with PRH that found *KRAS* mutations in 39% of CRC tumors.^40^ However, a similar *KRAS* mutational frequency was reported for USH (59%),^41^ while a higher frequency was reported for Mexicans (86%).^42^ In contrast, lower mutational frequencies have been reported for Caucasians (38%),^41^ Asians (37%),^41^ and African Americans (44.4%)^43^ in the United States, for Chileans (37%),^44^ and Brazilians (52%).^24^ CRC patients with tumors harboring *KRAS/NRAS* mutations do not respond to anti‐EGFR mAb therapy and have lower overall survival when compared to those with wild‐type tumors.^45^ Table 4 shows the current biomarkers used to guide CRC treatment, indicating that only patients with wild‐type *KRAS/NRAS* have the clinical benefit of anti‐EGFR mAb therapies. For PRH, 43.8% had a wild‐type *KRAS* status, whereas higher frequencies were observed for the TCGA, GENIE‐NH, and GENIE‐H (59.2%, 56.8%, and 53.3%, respectively). The majority of the missense mutations found in *KRAS* for PRH occur at exon 2 at codons 12 and 13, followed by exon 3 at codon 61 and exon 4 at codon 146. Our findings correspond with previous reports that the most common mutations for *KRAS* are found in exon 2 (70%–80% at codon 12 and 15%–20% at codon 13^46^), and least frequently in exon 3 at codon 61, all resulting in constitutively active GTPase increasing the downstream signaling pathway.^47^ PRH had a significantly higher frequency for the specific oncogenic mutations G12C, G13D, and Q61H when compared to the other datasets. Even though there is no actual treatment for CRC cases harboring *KRAS* mutations, several drugs have entered clinical trials targeting mutations at codons 12, 13, and 61 for mCRC. Some of these clinical trials are listed in Table 4. Recently, a drug called *sotarasib*, which targets the *KRAS* G12C mutant, received the FDA approval for treatment in non‐small cell lung cancer (NSCLC), and several investigational clinical trials are evaluating this drug for the treatment of mCRC patients harboring *KRAS* G12C and other mutations at codons 12 and 13. These clinical trials show promising benefits for PRH, who have significantly higher mutational frequencies of *KRAS* codon 12 and 13 mutations when compared to other populations. The high mutational burden of *KRAS* for PRH translates to a higher proportion of patients who cannot benefit from anti‐EGFR mAb therapies, could be correlated to the higher mortality rates observed for this population.

PRH had a lower *BRAF* mutational frequency when compared to TCGA and GENIE‐NH as well as to those previously reported for Hispanics (6%),^41^ Whites (12%),^41^ Asians (6%),^41^ and African Americans (6.4%)^48^ in the United States, and for Mexicans (6%).^42^ Even though *BRAF* an*d KRA*S mutations usually occur in a mutually exclusive manner,^49^ the biological consequence of these mutations is similar since both genes are part of the *EGFR/MAPK* signaling pathway. Patients harboring *BRAF* mutations have poorer outcomes when compared to their wild‐type counterparts, especially those carrying the V600E mutation (90% of *BRAF* alterations).^50^ Several studies have reported variable *BRAF* V600E mutational frequency (0%–15%) among Hispanics from Central and South America, with an average of 7.8%,^51^ higher than reported in this study for PRH (3.9%). Small molecule inhibitors targeting *BRAF* V600E in combination with anti‐EGFR targeted therapies have been demonstrated to improve overall survival in patients with mCRC carrying this mutation.^52^ As a result (see Table 4), the use of encorafenib, a BRAF kinase inhibitor, in combination with cetuximab (a mAb targeting EGFR), has been approved to treat mCRCs with *BRAF* V600E.

The frequency of *ERBB2* amplification for PRH was similar to that reported for the other studied datasets. *ERBB2* gene amplification represents 3%–4% of mCRCs and is associated with poor survival.^53^ The *ERBB2* V777L missense mutation was detected in only 0.6% of CRC tumors from PRH, which was lower compared to other datasets. Upregulation of *RAS/RAF/ERK* and *PI3K/PTEN/AKT* signaling pathways has been observed in CRC tumors having *ERBB2* gene amplification and/or V777L activating mutation.^54^, ^55^ Currently, targeted therapies with mAb against the ERBB2 receptor are the standard of care for mCRC tumors positive for *ERBB2* amplification. There are several ongoing clinical trials with immune checkpoint inhibitors, anti‐EGFR/ERBB2 mAb, and/or small molecule tyrosine kinase inhibitors for mCRC tumors harboring *ERBB2* activating mutations (see Table 4). Numerous studies have shown up‐regulation of ERBB2 signaling pathways as a mechanism of resistance for anti‐EGFR therapies in mCRC, which has led to consider *ERBB2* amplification/mutations as predictive biomarkers of resistance for anti‐EGFR therapies in *KRAS* and *BRAF* wild‐type mCRC.^56^

Although cancer outcomes are associated with ethnicity, the participation in clinical trials of individuals from diverse racial/ethnic groups remains substantially low (25.9%, 5.0%, 1.1%, 0.2%, and 0.9% for Whites, Asians, African Americans, Hispanics, and other minorities, respectively).^57^ Our team has examined the association between ancestral groups and CRC risk in PRH, showing that African American ancestry was associated with an increased risk of developing rectal tumors.^58^ This could partly explain the mutational frequency differences observed among PRH and other ethnic/racial groups, including Hispanic subgroups. For example, a lung cancer study performed with Latin Americans showed that Native American ancestry was strongly correlated with mutations in the *EGFR* and *KRAS* genes, underscoring the importance of providing genetic testing for Latin American patients with lung cancer with admixed ancestries.^59^ Future investigations are needed to assess ancestry‐specific germline variation among Hispanic CRC tumors. Efforts to increase the participation of ethnic/racial diverse populations in early‐phase clinical trials are of paramount importance to help elucidate the effect of germline variation as prognostic and predictive therapeutic biomarkers.

## 5 CONCLUSION

Some factors contributing to the observed differences in the mutational frequencies between PRH and the other datasets are secondary to differences in sample selection and methodologies used to assess mutations and ethnicity. Additional limitations of the present study are the limited clinical data on CRC tumors from PRH since the analysis was restricted to data of patients who underwent NGS in a clinical setting through commercial laboratory testing. Thus, there is limited information on socio‐demographic data, additional clinicopathologic variables, treatment regimens, and outcomes data. Nonetheless, this is the first study to systematically examine the somatic mutational profile and molecular alterations of PRH and contrast them with two large national and international datasets. Our study provides data on the specific mutational landscape for Hispanics with CRC and the implications on therapeutic options and clinical outcomes. Hence, future investigations into the carcinogenic pathways predominantly affecting Hispanic populations and the development of targetable therapies that will impact clinical outcomes among diverse populations are warranted.

The development of novel, comprehensive, and more accessible tumor interrogation methods, such as NGS, has paved the way for the identification of genomic drivers of cancer. Subsequently, this has improved our understanding of cancer health disparities for diverse populations, including underrepresented populations such as Hispanics. Our study provides the first comprehensive somatic molecular evaluation of colorectal tumors among PRH, USH, and non‐Hispanics demonstrating important differences in targetable biomarkers and carcinogenic pathways. The findings of this study will serve as a guide for future research and the identification of tailored cancer‐targeted therapies to improve overall CRC survival and decrease disparities among diverse populations.

## AUTHOR CONTRIBUTIONS

**Ingrid M. Montes‐Rodríguez:** Conceptualization (equal). **Hilmaris Centeno‐Girona:** Conceptualization (equal). **Camila Rivera Lynch:** Conceptualization (equal). **Norizda Rivera:** Conceptualization (equal). **Marcia Cruz‐Correa:** Conceptualization (equal).

## CONFLICT OF INTEREST STATEMENT

M.C.C. is a Board Member of the Precision Oncology Alliance. She receives research grants from Janssen, Genentech, Abbvie, Merck, Mirati, SeaGen, Pfizer, Taiho, Regeneron, Epigenomics, BMS, Gilead, and QED. I.M.M.R. declares no potential conflicts of interest. H.C.G. declares no potential conflicts of interest. C.R.L. declares no potential conflicts of interest. N.R. declares no potential conflicts of interest.

## ETHICS STATEMENT

This study was conducted in accordance with the guidelines of the Declaration of Helsinki, the Belmont Report, and the US Common Rule. In keeping with 45 CFR 46.101(b) (4), this study was performed utilizing retrospective, de‐identified clinical data. Therefore, this study is considered IRB‐exempt, and no patient consent was necessary from the subjects. This study was performed in strict accordance with the recommendations of data access guidelines of TCGA and AACR project GENIE datasets.

## Supporting information


Table S1.

Table S2.

## Data Availability

Supplementary tables with all the mutational data for PRH were included as a supplementary file (Table S2). The TCGA and GENIE datasets used for comparison analysis are publicly available in cBioportal: http://www.cbioportal.org and https://genie.cbioportal.org.
