# Supplementary material for: Molecular profiling of colorectal cancer in a genetically admixed Hispanic population
Source: Cancer Med. 2023 Apr 11;12(10):11686–702. doi: 10.1002/cam4.5888 (PMC10242354; doi:10.1002/cam4.5888)
Supplement: Supplementary file 1 — Table S1. [file CAM4-12-11686-s001.docx]

**Supplementary Table 1**. Emerging Biomarkers for CRC

| **Altered genes** | **Clinical Trial Therapies** | **Mechanism of action** |
| --- | --- | --- |
| *APC/ TP53/ PIK3CA* | Phase I - Toca 511+ Toca FC (NCT02576665) | - Toca 511: is a cancer-selective, retroviral replicating vector encoding yeast cytosine deaminase that converts 5-fluorocytosine (Toca-FC) into 5-fluorouracil which diffuses into the tumor microenvironment from Toca 511–infected cells. |
| *TP53* | Phase II - Lamivudine (NCT03144804) | - Lamivudine- a potent L1 reverse transcriptase (RT) inhibitor. |
|  | Phase II- TAK-931 (NCT03261947) | - TAK-931: an specific inhibitor of CDC7 kinase, which has specific roles in DNA replication and DNA damage response. |
|  | Phase II – Cyclophosphamide (NCT03149679) | - Cyclophosphamide: is an alkylating agent that upon absorption it is metabolized to phosphoramide mustard that could form DNA and RNA crosslinks irreversibly, leading to cell death. |
| *FBXW7/ PIK3CA* | Phase II - Everolimus (NCT01827384) | - Everolimus: is a Rapamycin analog that inhibits mTOR downstream of the PI3K/AKT pathway |
| *FBXW7* | Phase II - Berzosertib (NCT03718091) | - Berzosertib: is a potent and selective inhibitor of regulators of the DNA damage response pathway, ATM and ATR, which is active in cancer cells. |
|  | Phase II – Prexasertib (NCT02873975) | - Prexasertibit: inhibitor of CHK1 and CHK2, members of DNA damage response pathways. |
| *PIK3CA* | Phase I/II – CB-839 + Capecitabine (NCT02861300) | - CB-839: is a selective inhibitor of the glutaminase-1 enzyme, which convert glutamine into glutamate. Reduction of glutamate results in antineoplastic and pro-apoptotic activity in tumor cells. - Capecitabine (Xeloda): is an oral prodrug that is converted to its only active metabolite, Fluorouracil, in tumor cells, inhibiting DNA synthesis. |
|  | Phase I/II – MEN1611 + Cetuximab (NCT04495621) | - MEN1611: is a potent and selective small molecule inhibitor of PI3K targeting p110 α (both mutants and wild type) β and γ, while sparing δ subunit. |
|  | Phase II - Inavolisib (NCT04632992) | - Inavolisib: PIK3α inhibitor that selectively degrades mutant PI3Kα resulting in reduction of PI3K pathway activity. |
